# Performance of an Adipokine Pathway-Based Multilocus Genetic Risk Score for Prostate Cancer Risk Prediction

**DOI:** 10.1371/journal.pone.0039236

**Published:** 2012-06-29

**Authors:** Ricardo J. T. Ribeiro, Cátia P. D. Monteiro, Andreia S. M. Azevedo, Virgínia F. M. Cunha, Agnihotram V. Ramanakumar, Avelino M. Fraga, Francisco M. Pina, Carlos M. S. Lopes, Rui M. Medeiros, Eduardo L. Franco

**Affiliations:** 1 Molecular Oncology Group-CI, Portuguese Institute of Oncology, Porto, Portugal; 2 ICBAS, Abel Salazar Biomedical Sciences Institute, University of Porto, Porto, Portugal; 3 Division of Cancer Epidemiology, Department of Oncology, McGill University, Montreal, Canada; 4 LPCC–Portuguese League Against Cancer (NRNorte), Porto, Portugal; 5 Urology Department, D. Pedro V Military Hospital, Porto, Portugal; 6 Urology Department, S. João Hospital, Porto, Portugal; Baylor College of Medicine, United States of America

## Abstract

Few biomarkers are available to predict prostate cancer risk. Single nucleotide polymorphisms (SNPs) tend to have weak individual effects but, in combination, they have stronger predictive value. Adipokine pathways have been implicated in the pathogenesis. We used a candidate pathway approach to investigate 29 functional SNPs in key genes from relevant adipokine pathways in a sample of 1006 men eligible for prostate biopsy. We used stepwise multivariate logistic regression and bootstrapping to develop a multilocus genetic risk score by weighting each risk SNP empirically based on its association with disease. Seven common functional polymorphisms were associated with overall and high-grade prostate cancer (Gleason≥7), whereas three variants were associated with high metastatic-risk prostate cancer (PSA≥20 ng/mL and/or Gleason≥8). The addition of genetic variants to age and PSA improved the predictive accuracy for overall and high-grade prostate cancer, using either the area under the receiver-operating characteristics curves (P<0.02), the net reclassification improvement (P<0.001) and integrated discrimination improvement (P<0.001) measures. These results suggest that functional polymorphisms in adipokine pathways may act individually and cumulatively to affect risk and severity of prostate cancer, supporting the influence of adipokine pathways in the pathogenesis of prostate cancer. Use of such adipokine multilocus genetic risk score can enhance the predictive value of PSA and age in estimating absolute risk, which supports further evaluation of its clinical significance.

## Introduction

Prostate cancer is a complex and unpredictable disease, with risk being affected by advancing age, ethnic background and family history. Although the causes of prostate cancer are not yet fully understood, genetic variation influences disease risk [1]. Prostate cancer is usually accompanied by a rise in the concentration of serum PSA, which has been used for decades as a sensitive but poorly specific biomarker, and a controversial predictor of prostate cancer mortality [2], [3]. Many prostatic biopsies are unnecessary [4], which underscores the need for better prediction models with increased specificity to aid clinicians decide whether or not to recommend biopsy. Furthermore, this is especially relevant in men with mildly elevated PSA values (3–10 ng/mL), but where the risk for being diagnosed with prostate cancer is only about 20–25% [5]. After diagnosis, some cancers are indolent and cause no clinical problems, whereas others progress and may be fatal [6]. Therefore, it is important to search for biomarkers of aggressive clinical outcome. Genetic markers provide good candidates for such a role.

Single-nucleotide polymorphisms (SNPs) identified as loci associated with prostate cancer in genome-wide association studies (GWAS) are common but confer only small increases in risk and the mechanisms underlying their association with prostate cancer risk remain unknown [7], [8]. Recently, selected SNPs from GWAS were analyzed and converted into a genetic risk score, which was shown to reduce the number of biopsies although it did not discriminate aggressive cases [9].

The association between body mass and risk of prostate cancer is supported by meta-analyses that suggest increased risk of aggressive prostate cancer in the obese [10], and by studies using methods to estimate abdominal adiposity [11]. Recent work has focused on the role of adipokines and obesity-related molecules in the etiology of prostate cancer [12], [13]. Variants in genes encoding components of these pathways have been evaluated for prostate cancer risk and promising candidates have been identified [14], [15], [16], [17]. These candidate genes code for molecules found to be over- or under-expressed in obesity [18], [19], [20] and are involved in several biological mechanisms that modulate tumor proliferation, apoptosis, angiogenesis, motility, migration, and immunity [12], [21], i.e., traits that ultimately influence tumor behavior. Thus, common polymorphisms in adipokine pathways are plausible candidates that may help predict prostate cancer susceptibility. However, few studies have examined prostate cancer risk in the context of multi-loci SNPs in different adipokine pathways. In this report, we tested the hypothesis that SNPs in candidate genes involved in adipokine pathways may contribute to prostate cancer susceptibility and aggressiveness in a population of men referred for diagnostic surveillance. We also assessed the clinical utility of an adipokine genetic risk score to enhance the predictive value of age and PSA to predict high-risk individuals for screening and therapeutic management.

## Results

A total of 449 histologically confirmed prostate cancer and 557 non-prostate cancer patients were included in the analyses. Prostate cancer patients were older (P<0.0001) and presented with significantly higher circulating levels of PSA and a lower free/total PSA ratio (P<0.0001 and P<0.0001, respectively) (Table 1).

**Table 1:** Age and hormonal variables by disease status.

	Disease Status						
	Non-Prostate cancer			Prostate cancer			
	N^a^	Mean	Median	N^a^	Mean	Median	P^b^
Age, years	553	66.2	66.2	447	68.1	69.0	<0.0001
PSA, ng/mL	540	7.5	5.9	437	26.9	8.2	<0.0001
Free PSA, ng/mL	485	1.6	1.2	373	2.4	1.1	0.373
Free/Total PSA ratio	482	0.22	0.20	372	0.16	0.14	<0.0001
Serum Testosterone, ng/mL	494	478.0	444.5	381	471.5	443.0	0.690

a Number of evaluable patients for each variable;

b Differences between groups, Mann-Whitney test. PSA, prostate specific antigen.

We evaluated the associations between each individual SNP on prostate cancer susceptibility (Table S2). In the dominant effect models (referent: wild-type homozygote) there were significant decreases in risk for *LEPR* Gln223Arg (aOR = 0.6, 95%CI: 0.5–0.8, aOR = 0.6, 95%CI: 0.5–0.8 and aOR = 0.5, 95%CI: 0.4–0.8, for all, high-grade and high-risk prostate cancer for metastasis, respectively) and for *FGF2*+223 C>T (aOR = 0.7, 95%CI: 0.5–1.0 in high-grade prostate cancer). An increase in risk of high-grade prostate cancer was found in carriers of the *IL6R* Asp358Ala variant (aOR = 1.3, 95%CI: 1.0–1.7). In the recessive effect models (referent: wild-type homozygotes and heterozygotes) a significantly increased risk was observed for *IGF1R*+3174 G>A (aOR = 1.3, 95%CI: 1.0–1.9 for overall prostate cancer), *IGFBP3*-202 A>C (aOR = 1.3, 95%CI: 1.0–1.8 and aOR = 1.3, 95%CI: 1.0–1.8, for overall and high-grade prostate cancer, respectively) and with *SPP1*-66 T>C (aOR = 1.8, 95%CI: 1.1–3.0, aOR = 1.9, 95%CI: 1.1–3.2 and aOR = 2.4, 95%CI: 1.2–4.8, in overall, high-grade and high-risk prostate cancer for metastasis, respectively). Likewise, a significant protective effect for high-grade prostate cancer was observed for carriers of the *IL6*-597 G>A variant (aOR = 0.7, 95%CI: 0.4–1.0). Age-stratification on the aforementioned seven SNPs indicated that effects were mostly restricted to subjects below the median age (of non-cancer group, Table S3).

**Figure 1:**
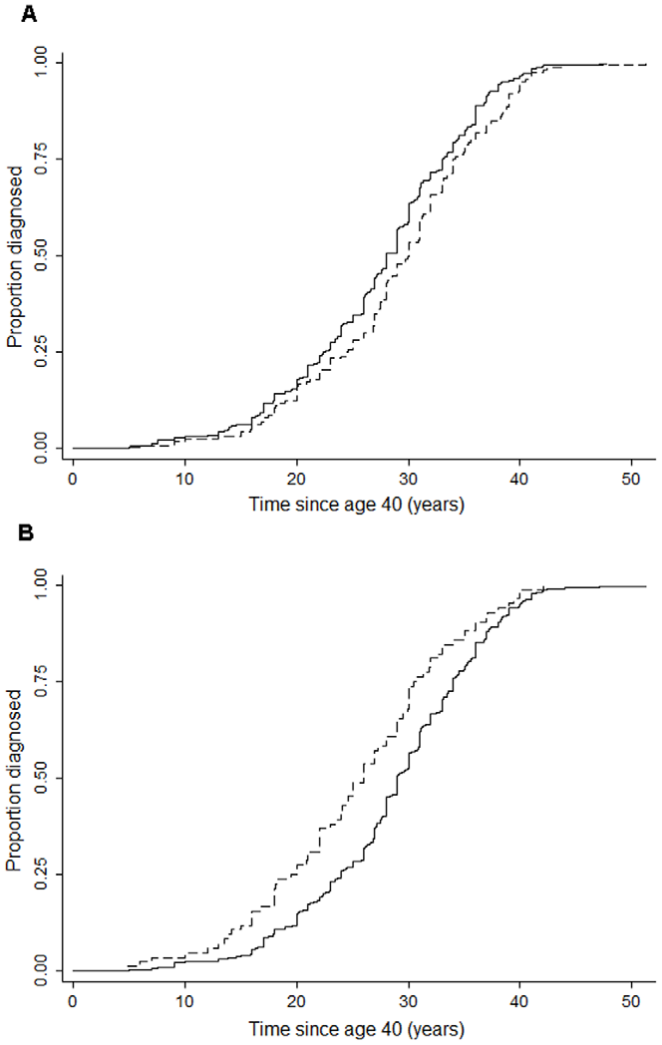
Kaplan Meier analyses plots of significant genetic polymorphisms. (A) *IL6R* D358A A>C and (B) *IGF1R*+3174 G>A. In figure 1A the dashed line corresponds to AA and the dotted line to CC/CA genotype. In figure 1B the dashed line represents AA, whereas the solid corresponds to GG/GA genotype. The Log Rank test was used to compare genotypes in *IL6R* D358A A>C (P = 0.026) and *IGF1R*+3174 G>A (P = 0.002).

Figure 1 shows that among prostate cancer cases there was a shorter waiting time-to-onset in *IL6R* Asp358Ala C-allele carriers (P = 0.026) and in *IGF1R*+3174 AA homozygous (P = 0.002). None of the other five SNPs influenced the time to onset of disease (data not shown).

To test our hypothesis that genetic variability in SNPs from adipokine pathways may contribute a combined effect for prostate cancer risk and/or aggressiveness, we estimated the overall mutually-adjusted effects by stepwise multivariate logistic regression. The SNPs in *LEPR* Gln223Arg, *SPP1*-66 T>G, *IGF1R*+3174 G>A, *IGFBP3*-202 A>C, *FGF2*+223 C>T and *IL6*-597 G>A, plus age and PSA remained independently associated with risk for overall, and for high-grade prostate cancer (Table 2). In the prostate cancer group with high risk for metastasis, only the *LEPR* Gln223Arg, *SPP1*-66 T>G and *FGF2*+223 C>T genetic variants, age and PSA persisted (Table 2). Within all groups, bootstrap analysis confirmed results (Table 2).

**Table 2:** Stepwise multivariate logistic regression and Bootstrap analyses.

		All PCa		Restricted to high-grade PCa		Restricted to high-risk PCa for Metastasis	
		Multivariate model	Bootstrap	Multivariate model	Bootstrap	Multivariate model	Bootstrap
	Genotype	OR (95%CI)^a^	OR (95%CI)^b^	OR (95%CI)^a^	OR (95%CI)^b^	OR (95%CI)^a^	OR (95%CI)^b^
Age at diagnosis		1.03 (1.01–1.05)	1.02 (1.00–1.04)	1.03 (1.01–1.05)	1.03 (1.01–1.06)	1.07 (1.03–1.11)	1.07 (1.03–1.11)
PSA at diagnosis		1.07 (1.04–1.09)	1.06 (1.04–1.09)	1.07 (1.05–1.10)	1.07 (1.04–1.11)	1.07 (1.04–1.09)	1.14 (1.09–1.19)
*LEPR* Gln223Arg	G carriers	Referent	Referent	Referent	Referent	Referent	Referent
(A>G)	AA	1.52 (1.14–2.02)	1.53 (1.13–2.07)	1.56 (1.15–2.12)	1.57 (1.14–2.14)	1.50 (0.91–2.45)	1.55 (0.93–2.58)
*SPP1*-66 T>G	T carriers	Referent	Referent	Referent	Referent	Referent	Referent
	GG	1.86 (1.07–3.23)	1.77 (1.00–3.13)	1.97 (1.10–3.52)	1.89 (1.03–3.49)	2.64 (1.16–6.01)	2.52 (1.12–5.64)
*IGF1R*+3174 G>A	G carriers	Referent	Referent	Referent	Referent		
	AA	1.33 (0.93–1.89)	1.34 (0.94–1.93)	1.40 (0.96–2.05)	1.39 (0.93–2.09)	–	–
*IGFBP3*-202 A>C	A carriers	Referent	Referent	Referent	Referent		
	CC	1.40 (1.02–1.92)	1.38 (1.01–1.88)	1.40 (1.00–1.95)	1.39 (1.00–1.93)	–	–
*FGF2*+223 C>T	T carriers	Referent	Referent	Referent	Referent	Referent	Referent
	CC	1.45 (0.98–2.14)	1.45 (0.98–2.16)	1.55 (1.00–2.38)	1.54 (1.00–2.38)	2.20 (1.01–4.78)	2.22 (1.02–4.85)
*IL6*-597 G>A	AA	Referent	Referent	Referent	Referent		
	G carriers	1.42 (0.92–2.19)	1.37 (0.88–2.13)	1.61 (0.99–2.62)	1.58 (0.97–2.56)	–	–

Age and PSA analyzed as continuous variables. PCa, prostate cancer. ^a^Stepwise multivariate logistic regression; ^b^MonteCarlo simulation (1000 replications). Empirical confounding variables were independently analyzed in each model (overall prostate cancer and both restricted groups).

The inclusive (age and PSA added to the multi-locus genetic set) linear risk scores computed on the basis of the above logistic regression models were tested as overall risk predictors categorized in tertiles based on the distribution in the non-prostate cancer group. As shown in Table 3, the risk for prostate cancer and high-grade prostate cancer increased according to the tertile of risk score (P_trend_ <0.0001 for both outcome categories). The age-adjusted ORs for unit changes in the inclusive risk score were 2.52 (95%CI: 2.0–3.2) and 2.77 (95%CI: 2.2–3.5) for all prostate cancers and high-grade prostate cancers, respectively. The goodness of fit for the logistic regression models based on the inclusive score were significantly greater than for the models based on the restricted age plus PSA score, for all prostate cancers (P = 0.0002) and high-grade prostate cancers (P = 0.0001), after likelihood ratio test.

**Table 3:** Tertiles of inclusive genetic risk score (GRS) and age-adjusted OR (CI 95%) for prostate cancer.

Inclusive Risk Score	Non-prostate cancer	All prostate cancer		High-grade prostate cancer	
Tertiles	N	N	aOR (95%CI)	N	aOR (95%CI)
T1	185	78	Referent	46	Referent
T2	186	101	1.2 (0.9–1.8)	85	1.7 (1.1–2.6)
T3	186	270	3.2 (2.3–4.6)	243	4.8 (3.2–7.2)

Tertiles for all prostate cancer: T1 (<2.74897), T2 (2.74897–3.15913), T3 (≥3.15913). Tertiles for high-grade prostate cancer: T1 (<2.85839), T2 (2.85839–3.30669), T3 (≥3.30669). The genetic risk scores were computed separately derived for overall and high-grade prostate cancer. aOR, age-adjusted ORs (95%CI).

**Figure 2:**
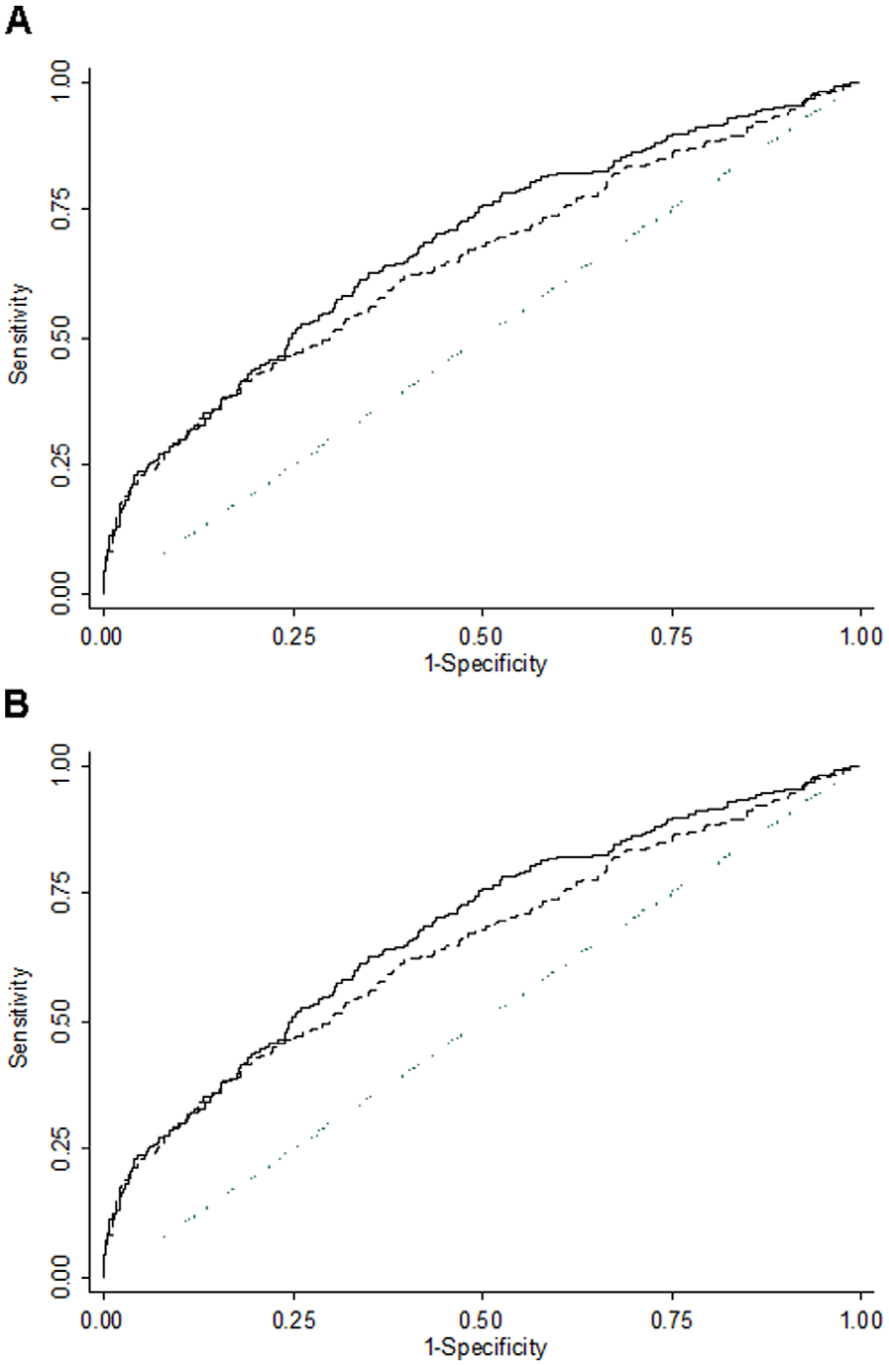
ROC curves and AUC for the inclusive risk score and PSA plus age alone. (A) All prostate cancer and (B) restricted to high-grade prostate cancer. Solid line corresponds to the all inclusive score, whereas dashed line represents the PSA and age risk score. The dotted line indicates the behavior of a hypothetical random score. The Likelihood ratio test was used to estimate the superiority of the inclusive risk score relative to that of the age+PSA score for all prostate cancer (inclusive: AUC = 0.6806, PSA and age: AUC = 0.6476, P = 0.0002) and high-grade prostate cancer (inclusive: AUC = 0.7119, PSA and age: AUC = 0.6808, P = 0.0001). PSA, prostate specific antigen.

Figure 2 shows the ROC curves for the all-inclusive genetic risk score and for the age and PSA-based risk score. The AUC estimates for both outcomes (all prostate cancers and high-grade prostate cancers) were significantly higher for the all-inclusive score than with the age plus PSA predictor, P = 0.0099 and P = 0.0196, respectively (Figure 2). The statistically superior predictive value of the all-inclusive score was confirmed via the NRI (all prostate cancers: 9.5%, P<0.0001, high-grade prostate cancer: 13.3%, P<0.0001) and IDI (all prostate cancers: 0.021, P<0.0001, high-grade prostate cancer: 0.024, P<0.0001) comparisons.

Genotype distributions in four SNPs deviated from Hardy-Weinberg equilibrium (Table S1). In sensitivity analysis of three relevant SNPs, equilibrium was achieved after restricting the control group to constrained conditions, whereas the trend towards increased risk remained stable, regardless of control group used (restricted or unrestricted) (Table S4). Three of these four deviated SNPs ended up in the all-inclusive risk score. Therefore, as an additional step to clarify the relative importance of these SNPs we tested a four SNP risk score (excluding the 3 SNPs that were not in equilibrium). Findings showed that the predictive and discriminative ability of the inclusive risk score based on 4 SNPs remained significant (data not shown). Therefore, we used the all inclusive score.

## Discussion

Adipose tissue deregulation has been proposed as a relevant mechanism underlying obesity-related cancer, due to inappropriate release of biologically active adipokines. Thus, functional SNPs in genes coding for molecules involved in adipokine pathways may modulate the expression, transport, or signaling of adipokines, thereby influencing prostate cancer risk and biology. Our findings show that SNPs in genes from adipokine pathways (leptin, interleukin-6, fibroblast growth factor 2, osteopontin, and insulin growth factor) may influence the development of prostate cancer and aggressive disease. Interestingly, we found that both the *LEPR* Gln223Arg homozygous A and *SPP1*-66 homozygous G were significantly associated with all outcomes (risks of overall, high-grade, and high metastatic-risk prostate cancers).

The pleiotrophic effects of leptin, namely in tumor development and progression are mediated by its receptor [12], [13]. Studies of SNPs affecting this pathway provided inconsistent results in prostate cancer. The leptin SNP at position -2548 was proposed as a susceptibility locus for prostate cancer [14], [15], albeit our data do not support this contention. Conversely, we found an increased risk in *LEPR* Gln223Arg homozygous A for prostate cancer, whereas others observed no such association [14], [16]. *LEPR* Gln223Arg AA carriers have lower leptin binding affinity to soluble leptin receptor and have increased circulating free leptin and soluble leptin receptor levels [22], [23]. Therefore, there is increased availability of leptin for binding to the long leptin receptor signaling isoform in the prostate tumor cell membrane. Cumulatively, the aminoacid change in this SNP may influence the signal for receptor intracellular recycling or degradation [24], modulating the availability of membrane-bound leptin receptor in tumor cells.

Osteopontin is a cytokine-like extracellular matrix molecule, that influences cell migration and anti-apoptosis in cancer [25]. This molecule has been implicated in aggressive and metastatic disease, and is one of a four-gene signature in prostate cancer that predicts metastasis and death [26], [27]. The T-to-G substitution at position -66 in the human *SPP1* gene modulates promoter activity [28]. The modified bioavailability of osteopontin may induce TH1-to-Th2 shift, modulating the microenvironment [28], and tumor development.

The IGF1-mediated activation of IGF1R has been demonstrated to contribute to tumor progression [29]. The IGF binding proteins modulate the effects of IGF1 and its biological function in different tissues. Recent evidence indicates increased risk of prostate cancer in individuals with high serum IGF1 levels, whereas risk was decreased in those with high levels of IGFBP-3 [30]. Furthermore, it was also found that the *IGFBP3*-202 A>C SNP was associated with prostate cancer and with low circulating levels of IGFBP3 [30]. The present study corroborates previous findings on the *IGFBP3*-202 A>C CC genotype risk for prostate cancer and high-grade disease [30], [31]. Cumulatively, functional studies confirmed the underexpression of *IGFBP3* in C-allele carriers [32], resulting in increased IGF1 bioavailability. Signaling through the IGF1R is required for growth and survival [29]. The synonymous *IGF1R* SNP at locus +3174 was described as a possible splicing regulator [33], thereby generating protein diversity [34] and serving as a mechanism for modulating gene expression [35]. Our findings showing that AA carriers remained independently associated with risk for all and for high-grade prostate cancer, suggest that this SNP may modulate IGF1R cell surface protein quantity, as well as IGF1R/IGF1R internalization and degradation, consequently influencing prostate tumor growth. Insulin receptor substrate –1 (IRS1) is the primary docking protein of IGF1R, which mediates PI3K pathway activation within the IGF1/IGF1R system. Although the *IRS1* Gly972Arg SNP results in structural protein differences [36] in our study this SNP was not associated with prostate cancer risk, confirming previous findings [37].

FGF2 may have a role in tumorigenesis and cancer progression through induction of angiogenesis [38]. The *FGF*+223 variant in exon 1 is associated with FGF2 expression at the transcriptional and translational level [39]. Our findings show increased risk for all, high-grade, and high-metastasis risk prostate cancer among CC carriers, which are coherent with a functional upregulation of FGF2. This molecule interacts with a family of four distinct, high-affinity tyrosine kinase receptors, FGFR 1–4. Although increased availability of FGF2 and changes in FGFR2 receptor availability could play a role in the initiation and progression of prostate cancer, we did not find an association between the *FGFR2* rs2981582 in exon 2 and prostate cancer.

Initiation and progression of prostate cancer are stimulated by IL-6 [40]. Previous findings reported no association of the *IL6*-174 G>C SNP with prostate cancer [17], [41], except for a small study of aggressive disease risk [42]. We did not find an association for the *IL6*-174 G>C SNP and prostate cancer. On the other hand, we found that carriers of the *IL6*-597 G-allele were at increased risk for high-grade prostate cancer. In fact, functional SNPs in the promoter region of *IL6* (-174, -572 and -597) do not act independently in the regulation of IL6 transcription [43]. The GG genotype in *IL6*-597 is linked to the GG genotype in *IL6*-174, which is associated with increased IL6 mRNA and protein levels. Therefore, the higher risk of high-grade prostate cancer associated with the *IL6*-597 G-allele may be due to increased IL6. IL6 signals are transmitted via a heterodimeric receptor complex consisting of a soluble interleukin-6 alpha subunit and a membrane-bound signal-transducing subunit, IL6ST. The common *IL6R* Asp385Ala variant is responsible for serum levels of soluble IL6R and IL6 and associates with IL6R membrane binding due to altered cleavage site [44], therefore, explaining our findings. The predominant activation of trans-signaling IL6/soluble IL6R pathway in aggressive prostate cancer [45], together with the functional IL6R Asp358Ala influence in this mechanism, supports the increased risk for high-grade prostate cancer we observed for C carriers (Ala carriers).

Several of the candidate SNPs in adipokine pathways known to affect oncogenesis, investigated here, were not associated with prostate cancer risk. Most of our null results for candidate SNPs in *ADIPOQ*+276, *VEGF*-460, *VEGF*+405, *VEGF*+936, *PPARG* Pro12Ala and *TNF*-308, are in agreement with other studies [14], [17], [46], [47]. To our knowledge, there have been no prior reports of null associations of *KDR*-604, *PPARD*-87, *PPARGC1A* Gly482Ser, *TNFRSF1A*-329, *ADIPOQ*+45, *ADIPOQ*-11426, *IL6ST* Gly148Arg, *IL6*-6331, and *TNF*-863 functional SNPs with prostate cancer.

We observed that some SNPs have a significant risk effect mainly in younger ages. The all-life exposure to increased levels of adipokines and pathway activation may influence early development of prostate cancer. Furthermore, *IL6R* Asp358Ala and *IGF1R* +3174 SNPs were significantly associated with early-onset prostate cancer, possibly due to accelerated tumor formation.

We tested each SNP for association with two clinically-relevant definitions of unfavorable outcomes: high-grade (combined Gleason score ≥7) and high-metastasis risk (combined Gleason score ≥8 and/or PSA≥20 ng/mL) prostate cancers. Combined Gleason score is a powerful predictor of disease progression and mortality [48], whereas Gleason score ≥8 is associated with aggressive biological behavior and increased risk of occult disseminated disease [49]. We found functional variants in genes from leptin, osteopontin, insulin growth factor, fibroblast growth factor 2 and interleukin 6 pathways to be related with high-grade prostate cancer, while SNPs in the leptin, osteopontin and fibroblast growth factor 2 axis associate with high-metastasis risk prostate cancer. These pathways are known to be involved in aggressive prostate cancer, lending support for these SNPs as clinical markers of aggressive disease. The SNPs in the risk score predict high grade/aggressive disease, but they also predict overall prostate cancer risk. The ability to predict overall as well as high grade cancers might be due to the significant proportion of high grade prostate cancer (Gleason≥7) (83%) in our cancer population.

Although a wealth of evidence demonstrates the effects of individual adipokines on prostate carcinogenesis, it is unlikely that the overall pathophysiological impact is due to the influence of a single adipokine in vivo. We showed that consideration of the cumulative susceptibility contributed by SNPs from adipokine pathways helps in risk stratification. Our analyses indicate that the inclusive (age and PSA added to the multi-locus genetic set) risk score provides improvements in discrimination and prediction of all prostate cancer, and high-grade prostate cancer. We suggest that risk genotypes in the inclusive model may cooperate to influence the endocrine and paracrine activity of adipokine pathways that leads to tumor development and progression. However, the mechanisms underlying these high-order interactions among genetic polymorphisms in adipokine pathways genes in modulating prostate cancer risk remain to be fully elucidated.

In this cohort of men subjected to prostate biopsy due to abnormal clinical and/or PSA findings where an extensive biopsy scheme was used, we showed that by adding a genetic score based on 7 SNPs significantly improved the discriminative ability of an established parsimonious model with only PSA and age. The AUC increased significantly from 0.65 to 0.68 for all prostate cancer and from 0.68 to 0.71 in high grade prostate cancer, when the genetic variants were added to the model. Furthermore, the improved predictive value of the score for prostate cancer risk persisted with a four SNPs risk score (excluding SNPs deviated from Hardy-Weinberg equilibrium). Although we present the largest effort to date to study the association between adipokine genetic risk score and risk of prostate cancer, our results should be interpreted in the context of several potential limitations. We took a focused candidate gene approach to evaluate key SNPs in adipokine pathways but our SNP panel could be incomplete. Likewise, several newly reported prostate cancer risk-associated SNPs from genome-wide association studies were not included in the risk prediction model. Had we been able to include them, the overall risk prediction might have improved. We also estimated risk associations in this study population with an exploratory intent, without having the opportunity to validate our findings in a separate sample of patients undergoing prostate cancer screening. Therefore, further studies in independent populations are required. Finally, despite our relatively large sample size, we had limited statistical power to examine genetic variants in relation to high-metastasis risk prostate cancer, because of the small number of cases in this group. However, our study has several strengths: i) it was prospective and large enough for key outcomes of interest, ii) most of the genes and SNPs selected were based on biological evidence of functional importance; iii) study design and statistical analyses accounted for relevant risk factors such as ethnicity and age [50], and although we did not have data on heredity information in a large set of subjects, only 2.2% were actually younger than 55 years of age, suggesting that hereditary prostate cancers were rare in our sample; iv) we used statistical strategies to assess the robustness of associations, such as bootstrap resampling and discrimination improvement measures; and v) all men were screened for prostate cancer based on both PSA level and digital rectal exam during the recruitment period and diagnosis was determined by standard biopsy, thus making outcome misclassification unlikely.

In summary, we identified SNPs in adipokine pathways that are associated with prostate cancer development and with a more aggressive phenotype. The inclusion of SNPs in the risk score model significantly improved, albeit modestly, the performance of PSA and age to predict overall prostate cancer and high-grade prostate cancer risk in men subjected to biopsy. The inclusion of further functional SNPs in a susceptibility model for prostate cancer is warranted, in order to determine a multi-locus model to accurately predict prostate cancer and disease aggressiveness. The use of improved risk models, such as the one described here, may impact public health strategies if shown to have clinical utility when combined with individualized screening and risk reduction strategies.

## Materials and Methods

### Ethics Statement

This study was approved by the ethics committees of Porto Military Hospital and São João Hospital (Porto, Portugal). Patients were included after signing a written informed consent.

### Subjects

Participants were enrolled between September 2007 and October 2010, after being referred to the urology departments of the participating hospitals for prostatic transrectal ultrasound guided biopsy (8–13 cores), on the basis of abnormal digital rectal examinations and/or single baseline PSA levels over 2.5 ng/mL. Our study population consisted of 1099 consecutively-admitted Caucasian men who had histological evaluation and consented for genotyping.

We selected a control group of patients with non-prostate cancer (benign prostate hyperplasia [BPH] or chronic prostatitis) from the prospectively enrolled men undergoing prostate biopsy. Our choice of this control group was based on the following reasons: (i) diagnosis was contemporary with that of cancers; (ii) their advanced age at diagnosis allowed matching with elderly cancer patients; (iii) all patients underwent digital rectal examination, PSA testing and prostate needle biopsy, making the possibility of crossover remote. Most men develop BPH or chronic prostatitis by the 7^th^–8^th^ decades of life, making it normal in men of that age to carry benign prostatic disease. This permitted our control group subjects to have comparable ages to those of our prostate cancer patients, thus minimizing the likelihood of outcome misclassification. Had we restricted controls to men without prostatic disease there would have been a severe imbalance in age distributions, which would introduce bias.

Prostate pathology and Gleason scores were determined via biopsy. In re-biopsed individuals only the last, most relevant pathological diagnosis was considered. Ninety-three men were excluded from the study due to a pathology report of high-grade prostatic intraepithelial neoplasia or a biopsy suspicious of cancer only. None of the participants had undergone prostate cancer treatment (hormonal castration, surgery, chemotherapy, or radiotherapy). All remaining 1006 eligible patients were included for molecular analysis.

### Genetic Variants and Genotyping

Candidate SNPs were selected from the best evidence from published studies and through public databases that provide information on the phenotypic risks. Candidate genes involved in adipokine pathways known to affect oncogenesis were selected. SNPs with minor allele frequencies <0.05 were excluded. A total of 29 literature-defined putative functional SNPs in 19 different genes were selected, corresponding to 9 adipokine pathways (Table S1).

Genotyping for 22 SNPs (two in *ADIPOQ*, *IL6*, *IL6R*, *KDR,* three in *VEGF*, *LEP*, two in *LEPR*, *PPARG*, *PPARGC1A*, *PPARD*, *SPP1*, *IGF1R*, *IGFBP3*, *IRS1*, *FGF2*, *FGFR2*, *TNF*, *TNFRSF1A*) was performed using TaqMan allelic discrimination (Applied Biosystems), whereas 7 SNPs were genotyped through polymerase chain reaction - restriction fragment length polymorphism analysis (*IL6*-597/−572/−174, *ADIPOQ*+45, *IL6ST* Gly148Arg, *LEPR* Gln223Arg and *TNF*-863), using previously described protocols. For quality control we used non-template controls in all runs and blind replicate genotype assessment in 5% of the samples. For the majority of SNPs, we observed almost complete concordance among duplicates.

### Statistical Analysis

The Mann-Whitney test was used to compare means between prostate cancer and non-cancer groups. The chi-square test was used to test for departures from Hardy-Weinberg equilibrium for each SNP based on the distribution among the non-prostate cancer group.

Unconditional logistic regression was used to estimate age-adjusted odds ratios (aORs) and 95% confidence intervals (95%CIs) for the associations between the polymorphisms and development of prostate cancer based on both recessive and dominant models. We examined the association of genetic markers with overall prostate cancer, restricted to high-grade prostate cancer (combined Gleason score ≥7), and restricted to high-risk prostate cancer for metastasis (PSA at diagnosis ≥20 ng/mL and/or combined Gleason score ≥8). Sensitivity analyses were conducted on the risk-associated SNPs that exhibited deviation from Hardy-Weinberg equilibrium. This was done by restricting the non-prostate cancer group to normal/BPH histology, and with serum PSA <4 ng/mL and then retesting the risk associations and departure from Hardy-Weinberg equilibrium.

To assess whether risk-associated SNPs affected time to clinical onset of disease we constructed Kaplan-Meier plots of the cumulative probabilities for having prostate cancer diagnosed at different ages according to each SNP. This analysis was conducted among prostate cancer cases only.

Stepwise multivariate logistic regression with backward elimination (P-value for retention = 0.15) was conducted in SNPs with aOR ≤0.7 or aOR ≥1.3 (30% decrease or increase in odds of the outcome) plus age and PSA as continuous variables. Bootstrapping analyses were performed through MonteCarlo simulation (1000 replications).

We constructed an inclusive multi-locus genetic risk score for each participant by summing the coefficients for each of the resulting variables after stepwise regression analyses. For each SNP, the risk genotypes were coded as 1 and the non-risk alleles as 0. The model was determined by multiplying the β coefficient by the SNPs, plus the γ coefficient by the PSA value and the α coefficient by the patient’s age (Inclusive Risk Score  = Σ βi x Xi+γ x PSA+α x Age; where Xi = SNPs scaled for risk, βi = coefficient for SNPs, γ = coefficient for PSA, α = coefficient for Age). A parsimonious risk score was calculated based on a model that included only PSA and age at diagnosis. These models were fitted independently using all prostate cancers and then restricted to high-grade prostate cancers as outcomes. A likelihood-ratio test was used to assess the goodness of fit between the two logistic regression models.

We assessed the clinical value of the above two scores in correctly predicting disease status by receiver operating characteristic (ROC) curve analysis. We compared the areas under the ROC curves (AUC) constructed with both scores (with and without genetic information), both for all prostate cancers and high-grade cancers, using a non-parametric algorithm [51].

We evaluated the improvement in model performance (PSA and age risk score) introduced by the inclusion of the SNPs risk information, using the net reclassification improvement (NRI) and the integrated discrimination improvement (IDI) tests [52], [53]. The NRI measures the reclassification of men from one risk category to another by addition of the genetic information to the PSA and age prediction model, and the extent of clinical utility can be evaluated by the magnitude of the NRI. The IDI does not consider risk thresholds; rather it is the mean of increments and decrements in estimated probabilities of prostate cancer for cases and non cases, comparing models. Since the NRI measurement is heavily dependent on the threshold levels used, we used a threshold probability between 15% and 45%, similar to those previously reported in such clinical context [54].

All statistical analyses were conducted in STATA version 10.0 (StataCorp, College Station, Texas). For NRI and IDI calculations, we used the nriidi-package for Stata 11 [53].

## Supporting Information

Table S1 **Characteristics of candidate Single Nucleotide Polymorphisms (SNPs) involved in adipokine pathways potentially associated with cancer.** HW-E, Hardy-Weinberg Equilibrium; *ADIPOQ*, adiponectin gene; *IL6*, interleukin-6 gene; *IL6R*, interleukin-6 receptor gene; *IL6ST*, interleukin-6 signal transducer gene; *KDR*, vascular endothelial growth factor receptor 2 gene; *VEGF*, vascular endothelial growth factor gene; *LEP*, leptin gene; *LEPR*, leptin receptor gene; *PPARGC1A*, Peroxisome proliferator-activated receptor gamma co-activator 1 alpha gene; *PPARD*, Peroxisome proliferator-activated receptor delta gene; *PPARG*, Peroxisome proliferator-activated receptor gamma gene; *SPP1*, osteopontin gene; *IRS1*, insulin receptor substrate 1 gene; *IGFBP3*, insulin growth factor binding protein 3 gene; *IGF1R*, insulin growth factor 1 receptor gene; *FGF2*, fibroblast growth factor 2 gene; *FGFR2*, fibroblast growth factor receptor 2 gene; *TNF*, tumoral necrosis factor alpha gene; *TNFRSF1A*, tumoral necrosis factor receptor 1 gene. ^a^ The percentage of successfully genotyped DNA samples from the 1006 participants.
(DOC)

Table S2 **Age-adjusted Odds Ratios and 95%CI of prostate cancer (PCa) according to adipokine pathways polymorphisms.** N, number of evaluable patients; SNP, single nucleotide polymorphism; OR (95%CI), age-adjusted odds-ratio and respective 95% confidence interval. ^a^ HGPCa,High-grade Prostate Cancer (Gleason grade ≥7). ^b^ HRPCaM, High-risk Prostate Cancer for metastasis (Gleason grade ≥8 and/or PSA ≥20 ng/mL).
(DOC)

Table S3 **Age-adjusted Odds Ratios and 95%CI for prostate cancer (PCa) associated with selected** ***SNPs,*** **after age stratification.** ^a^ High-grade Prostate Cancer, Gleason grade ≥7; ^b^ High-risk Prostate Cancer for metastasis, Gleason grade ≥8 and/or PSA ≥20 ng/mL; aOR (95%CI), age-adjusted odds ratio and respective 95% Confidence Interval; PCa, Prostate Cancer; Median age at diagnosis = 67.5 years; *Evaluable individuals for analysis.
(DOC)

Table S4 **Sensitivity analysis in SNPs with deviation from Hardy-Weinberg equilibrium.** Risk for prostate cancer after restriction on the non-prostate cancer group to just benign prostate hyperplasia and normal or to PSA below 4 ng/mL. *Hardy-Weinberg equilibrium, Pearson chi-square analysis for differences between observed and expected genotype frequencies; **Age-adjusted odds ratios; BPH, Benign Prostate Hyperplasia; PSA, Prostate-specific Antigen; PSA, prostate-specific antigen; SNP, signle nucleotide polymorphism; aOR (95%CI), age-adjusted odds ratio and respective 95% confidence interval. ^a^ Biopsy findings: normal, 14.9%; BPH, 5.4%, chronic prostatitis, 74.7%; atrophy, 5%; ^b^ Biopsy findings: normal, 73.5%; BPH, 26.5%; ^c^ Biopsy findings: normal, 22.2%; BPH, 6.0%, chronic prostatitis, 65.8%; atrophy, 6.0%.
(DOC)
