# Supplementary material for: Performance of an Adipokine Pathway-Based Multilocus Genetic Risk Score for Prostate Cancer Risk Prediction
Source: PLoS One. 2012 Jun 29;7(6):e39236. doi: 10.1371/journal.pone.0039236 (PMC3387135; doi:10.1371/journal.pone.0039236)
Supplement: Table S1 — Characteristics of candidate Single Nucleotide Polymorphisms (SNPs) involved in adipokine pathways potentially associated with cancer. HW-E, Hardy-Weinberg Equilibrium; ADIPOQ, adiponectin gene; IL6, interleukin-6 gene; IL6R, interleukin-6 receptor gene; IL6ST, interleukin-6 signal transducer gene; KDR, vascular endothelial growth factor receptor 2 gene; VEGF, vascular endothelial growth factor gene; LEP, leptin gene; LEPR, leptin receptor gene; PPARGC1A, Peroxisome proliferator-activated receptor gamma co-activator 1 alpha gene; PPARD, Peroxisome proliferator-activated receptor delta gene; PPARG, Peroxisome proliferator-activated receptor gamma gene; SPP1, osteopontin gene; IRS1, insulin receptor substrate 1 gene; IGFBP3, insulin growth factor binding protein 3 gene; IGF1R, insulin growth factor 1 receptor gene; FGF2, fibroblast growth factor 2 gene; FGFR2, fibroblast growth factor receptor 2 gene; TNF, tumoral necrosis factor alpha gene; TNFRSF1A, tumoral necrosis factor receptor 1 gene. a The percentage of successfully genotyped DNA samples from the 1006 participants. (DOC) [file pone.0039236.s001.doc]

Table S1. Characteristics of candidate Single Nucleotide Polymorphisms (SNPs) involved in adipokine pathways potentially associated with cancer

|  |  |  |  |  |  | In controls | |
| --- | --- | --- | --- | --- | --- | --- | --- |
| Pathway | Gene | Reference SNP ID | Nucleotide substitution | Genomic location | Genotyped (%) ^a^ | Variant allele  (%) | HW-E  (P-value) |
| Adiponectin | *APM1*  *APM1*  *APM1* | rs1501299  rs2241766  rs16861194 | G>T  T>G  A>G | + 276 intron 2  +45 intron 2  -11426 promoter | 99.8  100  100 | 29.9  13.9  10.4 | 0.80  0.91  1.00 |
| Interleukin - 6 | *IL6*  *IL6*  *IL6*  *IL6*  *IL6R*  *gp130* | rs1800795  rs1800796  rs1800797  rs10499563  rs2228145  rs3729960 | G>C  G>C  G>A  T>C  A>C  G>C | -174 promoter  -572 promoter  -597 promoter  -6331 promoter  Asp358Ala  Gly148Arg | 99.8  99.9  99.9  100  99.9  99.9 | 36.2  14.3  35.3  27.5  36.9  9.5 | 0.98  0.89  0.89  0.91  0.62  0.63 |
| Vascular Endothelial Growth Factor | *KDR*  *VEGF*  *VEGF*  *VEGF* | rs2071559  rs2010963  rs833061  rs3025039 | T>C  G>C  C>T  C>T | -604 promoter  +405 5’-UTR  -460 promoter  +936 3’-UTR | 100  99.8  99.8  99.8 | 47.1  32.3  48.2  13.1 | 0.96  0.72  0.95  0.85 |
| Leptin | *LEP*  *LEPR*  *LEPR*  *LEPR* | rs7799039  rs1137100  rs1137101  rs8179183 | G>A  A>G  A>G  G>C | -2548 promoter  Lys109Arg  Gln223Arg  Lys656Asn | 99.8  99.8  100  99.8 | 39.3  22.8  44.9  20.3 | 0.96  0.77  < 0.01  0.30 |
| Peroxisome proliferator-activated receptor | *PPARGC1A*  *PPARD*  *PPARG* | rs8192678  rs2016520  rs1801282 | A>G  T>C  C>G | Gly482Ser  -87 5’-UTR  Pro12Ala | 99.7  99.7  99.9 | 37.3  21.4  8.8 | 0.83  0.93  0.37 |
| Osteopontin | *OPN* | rs28357094 | T>G | -66 promoter | 99.9 | 22.8 | 0.97 |
| Insulin growth factor 1 | *IRS1*  *IGFBP3*  *IGF1R* | rs1801278  rs2854744  rs2229765 | C>T  A>C  G>A | Gly972Arg  -202 promoter  +3174 exon 16 | 100  100  100 | 9.7  47.3  43.7 | < 0.01  0.63  < 0.01 |
| Fibroblast growth factor 2 | *FGF2*  *FGFR2* | rs1449683  rs2981582 | C>T  C>T | +223 5’-UTR  Intron 2 | 99.7  100 | 9.5  36.7 | < 0.01  0.78 |
| Tumor necrosis factor alpha | *TNFA*  *TNFA*  *TNFRSF1A* | rs1800629  rs1800630  rs4149570 | G>A  C>A  G>T | -308 promoter  -863 promoter  -329 promoter | 100  99.8  99.9 | 14.1  21.1  38.8 | 0.36  0.19  0.61 |

HW-E, Hardy-Weinberg Equilibrium; *APM1*, adiponectin gene; *IL6*, interleukin-6 gene; *IL6R*, interleukin-6 receptor gene; *GP130*, interleukin-6 signal transducer gene; *KDR*, vascular endothelial growth factor receptor 2 gene; *VEGF*, vascular endothelial growth factor gene; *LEP*, leptin gene; *LEPR*, leptin receptor gene; *PPARGC1A*, Peroxisome proliferator-activated receptor gamma co-activator 1 alpha gene; *PPARD*, Peroxisome proliferator-activated receptor delta gene; *PPARG*, Peroxisome proliferator-activated receptor gamma gene; *OPN*, osteopontin gene; *IRS1*, insulin receptor substrate 1 gene; *IGFBP3*, insulin growth factor binding protein 3 gene; *IGF1R*, insulin growth factor 1 receptor gene; *FGF2*, fibroblast growth factor 2 gene; *FGFR2*, fibroblast growth factor receptor 2 gene; *TNFA*, tumoral necrosis factor alpha gene; *TNFRSF1A*, tumoral necrosis factor receptor 1 gene. ^a^ The percentage of successfully genotyped DNA samples from the 1006 participants.
