# Supplementary material for: Performance of an Adipokine Pathway-Based Multilocus Genetic Risk Score for Prostate Cancer Risk Prediction
Source: PLoS One. 2012 Jun 29;7(6):e39236. doi: 10.1371/journal.pone.0039236 (PMC3387135; doi:10.1371/journal.pone.0039236)
Supplement: Table S2 — Age-adjusted Odds Ratios and 95%CI of prostate cancer (PCa) according to adipokine pathways polymorphisms. N, number of evaluable patients; SNP, single nucleotide polymorphism; OR (95%CI), age-adjusted odds-ratio and respective 95% confidence interval. a HGPCa,High-grade Prostate Cancer (Gleason grade ≥7). b HRPCaM, High-risk Prostate Cancer for metastasis (Gleason grade ≥8 and/or PSA ≥20 ng/mL). (DOC) [file pone.0039236.s002.doc]

Table S2. Age-adjusted Odds Ratios and 95%CI of prostate cancer (PCa) according to adipokine pathways polymorphisms

|  |  |  |  |  | Age-adjusted Odds Ratios | | | | | | | |
| --- | --- | --- | --- | --- | --- | --- | --- | --- | --- | --- | --- | --- |
|  |  |  |  | Non-PCa | All PCa | |  | Restricted to HGPCa ^a^ | |  | Restricted to HRPCaM ^b^ | |
| Pathway | SNP | Model | Genotypes | N | N | OR (95% CI) |  | N | OR (95% CI) |  | N | OR (95% CI) |
| Adiponectin | *APM1*+45 T>G |  | TT  TG  GG | 414  131  12 | 347  90  12 | Referent  0.8 (0.3-1.8)  0.9 (0.4-1.9) |  | 289  74  11 | Referent  0.7 (0.3-1.7)  0.8 (0.3-1.8) |  | 120  30  5 | Referent  0.7 (0.2-2.1)  0.7 (0.2-2.2) |
|  |  | Dominant | TT  G carriers | 414  143 | 347  102 | Referent  0.9 (0.7-1.2) |  | 289  85 | Referent  0.9 (0.7-1.3) |  | 120  35 | Referent  1.0 (0.6-1.5) |
|  |  | Recessive | T carriers  GG | 545  12 | 437  12 | Referent  1.2 (0.5-2.7) |  | 363  11 | Referent  1.3 (0.6-3.0) |  | 150  5 | Referent  1.4 (0.5-4.2) |
|  | *APM1*+276 G>T |  | GG  GT  TT | 276  226  53 | 228  172  49 | Referent  0.8 (0.5-1.3)  0.9 (0.6-1.4) |  | 196  138  40 | Referent  0.9 (0.5-1.4)  1.0 (0.6-1.6) |  | 79  58  18 | Referent  0.8 (0.4-1.6)  0.9 (0.5-1.7) |
|  |  | Dominant | GG  T carriers | 276  279 | 228  221 | Referent  1.0 (0.7-1.2) |  | 196  178 | Referent  0.9 (0.7-1.2) |  | 79  76 | Referent  1.0 (0.7-1.4) |
|  |  | Recessive | G carriers  TT | 502  53 | 400  49 | Referent  1.1 (0.7-1.7) |  | 334  40 | Referent  1.1 (0.7-1.7) |  | 137  18 | Referent  1.1 (0.6-2.0) |
|  | *APM1*-11426 A>G |  | AA  AG  GG | 447  104  6 | 360  85  4 | Referent  1.3 (0.4-4.8)  1.3 (0.4-4.6) |  | 299  71  4 | Referent  1.1 (0.3-4.1)  1.1 (0.3-3.9) |  | 123  31  1 | Referent  2.0 (0.2-18.0)  1.8 (0.2-15.6) |
|  |  | Dominant | AA  G carriers | 447  110 | 360  89 | Referent  1.0 (0.7-1.4) |  | 299  75 | Referent  1.0 (0.7-1.4) |  | 123  32 | Referent  1.1 (0.7-1.7) |
|  |  | Recessive | A carriers GG | 551  6 | 445  4 | Referent  0.6 (0.1-2.4) |  | 370  4 | Referent  0.7 (0.2-2.8) |  | 154  1 | Referent  0.5 (0.1-4.7) |
| Interleukine 6 | *IL6*-6331 T>C |  | TT  TC  CC | 291  226  40 | 236  185  28 | Referent  1.0 (0.8-1.3)  0.9 (0.5-1.5) |  | 198  153  23 | Referent  1.0 (0.8-1.3)  0.9 (05-1.5) |  | 87  60  8 | Referent  0.9 (0.6-1.4)  0.7 (0.3-1.6) |
|  |  | Dominant | TT  C carriers | 291  266 | 236  213 | Referent  1.0 (0.8-1.3) |  | 198  176 | Referent  1.0 (0.7-1.3) |  | 87  68 | Referent  0.9 (0.6-1.3) |
|  |  | Recessive | T carriers  CC | 517  40 | 421  28 | Referent  0.9 (0.5-1.4) |  | 351  23 | Referent  0.9 (0.5-1.5) |  | 147  8 | Referent  0.7 (0.3-1.7) |

Supplementary table II. Age-adjusted Odds Ratios and 95%CI of prostate cancer according to adipokine pathways polymorphisms (cont´d)

|  |  |  |  |  | Age-adjusted Odds Ratios | | | | | | | |
| --- | --- | --- | --- | --- | --- | --- | --- | --- | --- | --- | --- | --- |
|  |  |  |  | Non-PCa | All PCa | |  | Restricted to HGPCa ^a^ | |  | Restricted to HRPCaM ^b^ | |
| Pathway | SNP | Model | Genotypes | N | N | OR (95% CI) |  | N | OR (95% CI) |  | N | OR (95% CI) |
| Interleukine 6 | *IL6*-597 G>A |  | GG  GA  AA | 235  249  72 | 201  204  44 | Referent  1.0 (0.7-1.3)  0.7 (0.5-1.1) |  | 167  173  34 | Referent  1.0 (0.8-1.3)  0.7 (0.4-1.1) |  | 69  69  17 | Referent  1.0 (0.7-1.4)  0.8 (0.4-1.5) |
|  |  | Dominant | GG  A carriers | 235  321 | 201248 | Referent  0.9 (0.7-1.2) |  | 167207 | Referent  0.9 (0.7-1.2) |  | 69  86 | Referent  0.9 (0.6-1.4) |
|  |  | Recessive | G carriers AA | 484  72 | 40544 | Referent  0.7 (0.5-1.1) |  | 34034 | Referent  0.7 (0.4-1.0) |  | 13817 | Referent  0.8 (0.5-1.5) |
|  | *IL6*-572 G>C |  | GG  GC  CC | 407  139  10 | 314  125  10 | Referent  1.2 (0.9-1.6)  1.3 (0.5-3.2) |  | 259  106  9 | Referent  1.2 (0.9-1.7)  1.5 (0.6-3.7) |  | 109  45  1 | Referent  1.3 (0.9-2.0)  0.4 (0.0-3.0) |
|  |  | Dominant | GG  C carriers | 407  149 | 314  135 | Referent  1.2 (0.9-1.6) |  | 259  115 | Referent  1.2 (0.9-1.7) |  | 109  46 | Referent  1.2 (0.8-1.8) |
|  |  | Recessive | G carriers  CC | 546  10 | 439  10 | Referent  1.2 (0.5-3.0) |  | 365  9 | Referent  1.4 (0.6-3.5) |  | 154  1 | Referent  0.3 (0.0-2.8) |
|  | *IL6*-174 G>C |  | GG  GC  CC | 225  259  72 | 194  209  45 | Referent  0.9 (0.7-1.2)  0.7 (0.5-1.1) |  | 159  177  37 | Referent  1.0 (0.7-1.3)  0.7 (0.5-1.2) |  | 63  74  17 | Referent  1.0 (0.7-1.5)  0.8 (0.5-1.6) |
|  |  | Dominant | GG  C carriers | 225  331 | 194  254 | Referent  0.9 (0.7-1.2) |  | 159  214 | Referent  0.9 (0.7-1.2) |  | 63  91 | Referent  1.0 (0.7-1.4) |
|  |  | Recessive | G carriers  CC | 484  72 | 403  45 | Referent  0.8 (0.5-1.1) |  | 336  37 | Referent  0.7 (0.5-1.1) |  | 137  17 | Referent  0.8 (0.5-1.5) |
|  | *IL6R* Asp358Ala (A>C) |  | AA  AC  CC | 227  248  81 | 164  209  76 | Referent  1.2 (0.9-1.6)  1.3 (0.9-1.9) |  | 132  176  66 | Referent  1.2 (0.9-1.7)  1.4 (1.0-2.1) |  | 54  70  31 | Referent  1.3 (0.8-1.9)  1.6 (0.9-2.6) |
|  |  | Dominant | AA  C carriers | 227  329 | 164  285 | Referent  1.2 (0.9-1.6) |  | 132  242 | Referent  1.3 (1.0-1.7) |  | 54  101 | Referent  1.3 (0.9-2.0) |
|  |  | Recessive | A carriers  CC | 475  81 | 373  76 | Referent  1.2 (0.8-1.7) |  | 308  66 | Referent  1.3 (0.9-1.8) |  | 124  31 | Referent  1.4 (0.9-2.2) |

Supplementary table II. Age-adjusted Odds Ratios and 95%CI of prostate cancer according to adipokine pathways polymorphisms (cont´d)

|  |  |  |  |  | Age-adjusted Odds Ratios | | | | | | | |
| --- | --- | --- | --- | --- | --- | --- | --- | --- | --- | --- | --- | --- |
|  |  |  |  | Non-PCa | All PCa | |  | Restricted to HGPCa ^a^ | |  | Restricted to HRPCaM ^b^ | |
| Pathway | SNP | Model | Genotypes | N | N | OR (95% CI) |  | N | OR (95% CI) |  | N | OR (95% CI) |
| Interleukine 6 | gp130 Gly140Arg(G>C) |  | GG  GC  CC | 457  92  7 | 381  60  8 | Referent  0.8 (0.6-1.2)  1.2 (0.4-3.5) |  | 316  52  6 | Referent  0.9 (0.6-1.3)  1.1 (0.4-3.4) |  | 127  25  3 | Referent  1.1 (0.7-1.9)  1.2 (0.3-4.9) |
|  |  | Dominant | GG  C carriers | 457  99 | 38168 | Referent  0.8 (0.6-1.2) |  | 31658 | Referent  0.9 (0.6-1.3) |  | 12728 | Referent  1.1 (0.7-1.8) |
|  |  | Recessive | G carriers CC | 549  7 | 4418 | Referent  1.3 (0.5-3.6) |  | 3686 | Referent  1.2 (0.4-3.5) |  | 1523 | Referent  1.2 (0.3-4.8) |
| Vascular endothelial growth factor | KDR-604 T>C |  | TT  CT  CC | 154  281  122 | 127  215  107 | Referent  0.9 (0.7-1.2)  1.1 (0.7-1.5) |  | 105  177  92 | Referent  0.9 (0.7-1.3)  1.1 (0.8-1.6) |  | 48  72  35 | Referent  0.8 (0.5-1.3)  1.0 (0.6-1.6) |
|  |  | Dominant | TT  C carriers | 154  403 | 127  322 | Referent  1.0 (0.7-1.3) |  | 105  269 | Referent  1.0 (0.7-1.3) |  | 48  107 | Referent  0.9 (0.6-1.3) |
|  |  | Recessive | T carriers  CC | 435  122 | 342  107 | Referent  1.1 (0.8-1.5) |  | 282  92 | Referent  1.2 (0.9-1.6) |  | 120  35 | Referent  1.1 (0.7-1.7) |
|  | VEGF-460 C>T |  | CC  CT  TT | 131  274  151 | 114  201  133 | Referent  0.9 (0.6-1.2)  1.1 (0.8-1.5) |  | 99  166  108 | Referent  0.8 (0.6-1.1)  1.0 (0.7-1.5) |  | 46  74  34 | Referent  0.8 (0.5-1.2)  0.7 (0.4-1.1) |
|  |  | Dominant | TT  C carriers | 151  405 | 133  315 | Referent  0.9 (0.6-1.1) |  | 108  265 | Referent  0.9 (0.6-1.2) |  | 34  120 | Referent  1.2 (0.8-1.9) |
|  |  | Recessive | T carriers  CC | 425  131 | 334  114 | Referent  1.1 (0.8-1.4) |  | 274  99 | Referent  1.1 (0.8-1.5) |  | 108  46 | Referent  1.3 (0.9-2.0) |
|  | VEGF+405 G>C |  | GG  GC  CC | 251  252  54 | 200  197  50 | Referent  1.0 (0.8-1.3)  1.2 (0.8-1.9) |  | 169  162  41 | Referent  1.0 (0.7-1.3)  1.2 (0.8-1.9) |  | 77  66  10 | Referent  0.8 (0.6-1.2)  0.7 (0.3-1.4) |
|  |  | Dominant | GG  C carriers | 251  306 | 200  247 | Referent  1.0 (0.8-1.3) |  | 169  203 | Referent  1.0 (0.8-1.3) |  | 77  76 | Referent  0.8 (0.6-1.2) |
|  |  | Recessive | G carriers  CC | 503  54 | 397  50 | Referent  1.2 (0.8-1.9) |  | 331  41 | Referent  1.2 (0.8-1.9) |  | 143  10 | Referent  0.7 (0.4-1.5) |

Supplementary table II. Age-adjusted Odds Ratios and 95%CI of prostate cancer according to adipokine pathways polymorphisms (cont´d)

|  |  |  |  |  | Age-adjusted Odds Ratios | | | | | | | |
| --- | --- | --- | --- | --- | --- | --- | --- | --- | --- | --- | --- | --- |
|  |  |  |  | Non-PCa | All PCa | |  | Restricted to HGPCa ^a^ | |  | Restricted to HRPCaM ^b^ | |
| Pathway | SNP | Model | Genotypes | N | N | OR (95% CI) |  | N | OR (95% CI) |  | N | OR (95% CI) |
| Vascular endothelial growth factor | VEGF+936 C>T |  | CC  CT  TT | 421  123  11 | 341  100  8 | Referent  1.0 (0.7-1.3)  0.9 (0.3-2.2) |  | 282  87  5 | Referent  1.0 (0.7-1.4)  0.7 (0.2-2.0) |  | 114  39  2 | Referent  1.1 (0.7-1.6)  0.7 (0.1-3.2) |
|  |  | Dominant | CC  T carriers | 421  134 | 341108 | Referent  1.0 (0.7-1.3) |  | 28292 | Referent  1.0 (0.7-1.3) |  | 11441 | Referent  1.0 (0.7-1.6) |
|  |  | Recessive | C carriers TT | 544  11 | 4418 | Referent  0.9 (0.4-2.2) |  | 3695 | Referent  0.7 (0.2-2.0) |  | 1532 | Referent  0.7 (0.1-3.1) |
| Leptin | LEP-2548 G>A |  | GG  GA  AA | 203  268  84 | 164  212  73 | Referent  1.0 (0.8-1.3)  1.1 (0.7-1.5) |  | 135  178  61 | Referent  1.0 (0.8-1.4)  1.1 (0.7-1.6) |  | 58  75  22 | Referent  1.0 (0.7-1.6)  0.9 (0.5-1.7) |
|  |  | Dominant | GG  A carriers | 203  352 | 164  285 | Referent  1.0 (0.8-1.3) |  | 135  239 | Referent  1.0 (0.8-1.4) |  | 58  97 | Referent  1.0 (0.7-1.5) |
|  |  | Recessive | G carriers  AA | 471  84 | 376  73 | Referent  1.1 (0.8-1.5) |  | 313  61 | Referent  1.1 (0.7-1.5) |  | 133  22 | Referent  0.9 (0.5-1.6) |
|  | LEPR Lys109Arg (A>G) |  | AA  AG  GG | 334  190  32 | 264  157  28 | Referent  1.0 (0.8-1.4)  1.1 (0.6-1.9) |  | 214  138  22 | Referent  1.1 (0.9-1.5)  1.1 (0.6-1.9) |  | 91  57  7 | Referent  1.1 (0.7-1.6)  0.8 (0.3-1.9) |
|  |  | Dominant | AA  G carriers | 334  222 | 264185 | Referent  1.0 (0.8-1.4) |  | 214160 | Referent  1.1 (0.9-1.5) |  | 91  64 | Referent  1.0 (0.7-1.5) |
|  |  | Recessive | A carriers GG | 524  32 | 42128 | Referent  1.0 (0.6-1.8) |  | 35222 | Referent  1.0 (0.5-1.7) |  | 1487 | Referent  0.8 (0.3-1.8) |
|  | LEPR Gln223Arg (A>G) |  | AA  AG  GG | 151  312  94 | 166  206  77 | Referent  0.6 (0.5-0.8)  0.7 (0.5-1.1) |  | 141  173  60 | Referent  0.6 (0.4-0.8)  0.7 (0.5-1.0) |  | 63  72  20 | Referent  0.6 (0.4-0.8)  0.5 (0.3-0.8) |
|  |  | Dominant | AA  G carriers | 151  406 | 166283 | Referent  0.6 (0.5-0.8) |  | 141233 | Referent  0.6 (0.5-0.8) |  | 63  92 | Referent  0.5 (0.4-0.8) |
|  |  | Recessive | A carriers GG | 463  94 | 37277 | Referent  1.0 (0.7-1.4) |  | 31460 | Referent  0.9 (0.6-1.3) |  | 13520 | Referent  0.7 (0.4-1.1) |

Supplementary table II. Age-adjusted Odds Ratios and 95%CI of prostate cancer according to adipokine pathways polymorphisms (cont´d)

|  |  |  |  |  | Age-adjusted Odds Ratios | | | | | | | |
| --- | --- | --- | --- | --- | --- | --- | --- | --- | --- | --- | --- | --- |
|  |  |  |  | Non-PCa | All PCa | |  | Restricted to HGPCa ^a^ | |  | Restricted to HRPCaM ^b^ | |
| Pathway | SNP | Model | Genotypes | N | N | OR (95% CI) |  | N | OR (95% CI) |  | N | OR (95% CI) |
| Leptin | LEPR Lys656Asn (G>C) |  | GG  GC  CC | 347  192  17 | 298  133  17 | Referent  0.8 (0.6-1.1)  1.1 (0.5-2.2) |  | 245  113  15 | Referent  0.8 (0.6-1.1)  1.1 (0.6-2.3) |  | 99  48  7 | Referent  0.9 (0.6-1.3)  1.4 (0.5-3.5) |
|  |  | Dominant | GG  C carriers | 347  209 | 298  150 | Referent  0.8 (0.6-1.1) |  | 245  128 | Referent  0.9 (0.7-1.1) |  | 99  55 | Referent  0.9 (0.6-1.4) |
|  |  | Recessive | G carriers  CC | 539  17 | 431  17 | Referent  1.2 (0.6-2.3) |  | 358  15 | Referent  1.2 (0.6-2.5) |  | 147  7 | Referent  1.4 (0.6-3.6) |
| Peroxisome proliferator-activated | PPARG Pro12Ala (C>G) |  | CC  CG  GG | 466  84  7 | 384  61  3 | Referent  0.9 (0.6-1.3)  0.5 (0.1-2.0) |  | 319  53  2 | Referent  0.9 (0.6-1.3)  0.4 (0.1-2.1) |  | 129  26  0 | Referent  1.1 (0.7-1.8)  ---- |
| receptors |  | Dominant | CC  G carriers | 466  91 | 38464 | Referent  0.9 (0.6-1.2) |  | 31955 | Referent  0.9 (0.6-1.3) |  | 12926 | Referent  1.0 (0.6-1.6) |
|  |  | Recessive | C carriers GG | 550  7 | 4453 | Referent  0.5 (0.1-2.1) |  | 3722 | Referent  0.4 (0.1-2.1) |  | 1550 | Referent  ---- |
|  | PGC1A Gly482Ser (A>G) |  | AA  AG  GG | 222  254  81 | 166  217  63 | Referent  1.2 (0.9-1.5)  1.1 (0.7-1.6) |  | 135  185  53 | Referent  1.3 (0.9-1.7)  1.1 (0.8-1.7) |  | 55  74  25 | Referent  1.2 (0.8-1.8)  1.3 (0.8-2.3) |
|  |  | Dominant | AA  G carriers | 222  335 | 166  280 | Referent  1.1 (0.9-1.5) |  | 135  238 | Referent  1.2 (0.9-1.6) |  | 55  99 | Referent  1.2 (0.8-1.8) |
|  |  | Recessive | A carriers  GG | 476  81 | 383  63 | Referent  1.0 (0.7-1.4) |  | 320  53 | Referent  1.0 (0.7-1.5) |  | 129  25 | Referent  1.2 (0.7-2.0) |
|  | PPARD -87 T>C |  | TT  TC  CC | 342  190  24 | 260  159  28 | Referent  1.1 (0.8-1.4)  1.5 (0.8-2.6) |  | 219  131  24 | Referent  1.1 (0.8-1.4)  1.5 (0.8-2.7) |  | 94  51  10 | Referent  1.0 (0.7-1.4)  1.4 (0.6-3.2) |
|  |  | Dominant | TT  C carriers | 342  214 | 260  187 | Referent  1.1 (0.9-1.5) |  | 219  155 | Referent  1.1 (0.8-1.5) |  | 94  61 | Referent  1.0 (0.7-1.5) |
|  |  | Recessive | T carriers  CC | 532  24 | 419  28 | Referent  1.4 (0.8-2.5) |  | 350  24 | Referent  1.5 (0.8-2.7) |  | 145  10 | Referent  1.5 (0.7-3.2) |

Supplementary table II. Age-adjusted Odds Ratios and 95%CI of prostate cancer according to adipokine pathways polymorphisms (cont´d)

|  |  |  |  |  | Age-adjusted Odds Ratios | | | | | | | |
| --- | --- | --- | --- | --- | --- | --- | --- | --- | --- | --- | --- | --- |
|  |  |  |  | Non-PCa | All PCa | |  | Restricted to HGPCa ^a^ | |  | Restricted to HRPCaM ^b^ | |
| Pathway | SNP | Model | Genotypes | N | N | OR (95% CI) |  | N | OR (95% CI) |  | N | OR (95% CI) |
| Osteopontin | OPN -66 T>G |  | TT  TG  GG | 330  198  28 | 268  143  38 | Referent  0.9 (0.7-1.2)  1.7(1.0-2.9) |  | 224  118  32 | Referent  0.9 (0.6-1.2)  1.8 (1.0-3.1) |  | 94  45  16 | Referent  0.8 (0.5-1.2)  2.2 (1.1-4.5) |
|  |  | Dominant | TT  G carriers | 330  226 | 268  181 | Referent  1.0 (0.8-1.3) |  | 224  150 | Referent  1.0 (0.7-1.3) |  | 94  61 | Referent  0.9 (0.7-1.4) |
|  |  | Recessive | T carriers  GG | 528  28 | 411  38 | Referent  1.8 (1.1-3.0) |  | 342  32 | Referent  1.9 (1.1-3.2) |  | 139  16 | Referent  2.4 (1.2-4.8) |
| Insulin growth factor | IGF1R+3174 G>A |  | GG  GA  AA | 158  311  88 | 133  232  84 | Referent  0.9 (0.7-1.2)  1.2 (0.8-1.8) |  | 111193  70 | Referent  0.9 (0.6-1.2)  1.2 (0.8-1.8) |  | 49  80  26 | Referent  0.9 (0.6-1.3)  1.1 (0.6-2.0) |
|  |  | Dominant | GG  A carriers | 158  399 | 133  316 | Referent  1.0 (0.7-1.3) |  | 111  263 | Referent  0.9 (0.7-1.3) |  | 49  106 | Referent  0.9 (0.6-1.4) |
|  |  | Recessive | G carriers  AA | 469  88 | 365  84 | Referent  1.3 (1.0-1.9) |  | 304  70 | Referent  1.3 (0.9-1.9) |  | 129  26 | Referent  1.2 (0.8-2.0) |
|  | IGFBP3-202 A>C |  | AA  AC  CC | 149  289  119 | 117  212  120 | Referent  0.9 (0.7-1.3)  1.3 (0.9-1.8) |  | 96  176  102 | Referent  1.0 (0.7-1.3)  1.3 (0.9-1.9) |  | 44  74  37 | Referent  0.9 (0.6-1.4)  1.0 (0.6-1.7) |
|  |  | Dominant | AA  C carriers | 149  408 | 117  332 | Referent  1.0 (0.8-1.4) |  | 96  278 | Referent  1.1 (0.8-1.4) |  | 44  111 | Referent  0.9 (0.6-1.4) |
|  |  | Recessive | A carriers  CC | 438  119 | 329  120 | Referent  1.3 (1.0-1.8) |  | 172  102 | Referent  1.3 (1.0-1.8) |  | 118  37 | Referent  1.1 (0.7-1.7) |
|  | IRS1 Gly972Arg (C>T) |  | CC  CT  TT | 461  84  12 | 374  68  7 | Referent  1.1 (0.7-1.4)  0.8 (0.3-2.0) |  | 314  54  6 | Referent  0.9 (0.6-1.4)  0.8 (0.3-2.2) |  | 130  21  4 | Referent  0.8 (0.5-1.4)  1.4 (0.4-4.7) |
|  |  | Dominant | CC  T carriers | 461  96 | 37475 | Referent  1.0 (0.7-1.3) |  | 31460 | Referent  0.9 (0.6-1.3) |  | 13025 | Referent  0.9 (0.5-1.5) |
|  |  | Recessive | C carriers TT | 545  12 | 4427 | Referent  0.8 (0.3-2.0) |  | 3686 | Referent  0.8 (0.3-2.2) |  | 1514 | Referent  1.5 (0.4-4.8) |

Supplementary table II. Age-adjusted Odds Ratios and 95%CI of prostate cancer according to adipokine pathways polymorphisms (cont´d)

|  |  |  |  |  | Age-adjusted Odds Ratios | | | | | | | |
| --- | --- | --- | --- | --- | --- | --- | --- | --- | --- | --- | --- | --- |
|  |  |  |  | Non-PCa | All PCa | |  | Restricted to HGPCa ^a^ | |  | Restricted to HRPCaM ^b^ | |
| Pathway | SNP | Model | Genotypes | N | N | OR (95% CI) |  | N | OR (95% CI) |  | N | OR (95% CI) |
| Fibroblast growth factor 2 | FGF2+223 C>T |  | CC  CT  TT | 463  80  13 | 391  52  4 | Referent  0.8 (0.5-1.2)  0.4 (0.1-1.2) |  | 328  41  3 | Referent  0.8 (0.5-1.1)  0.3 (0.1-1.2) |  | 138  15  1 | Referent  0.7 (0.5-1.3)  0.3 (0.0-2.3) |
|  |  | Dominant | CC  T carriers | 463  93 | 39156 | Referent  0.7 (0.5-1.1) |  | 32844 | Referent  0.7 (0.5-1.0) |  | 13816 | Referent  0.6 (0.4-1.2) |
|  |  | Recessive | C carriers TT | 543  13 | 4434 | Referent  0.4 (0.1-1.2) |  | 3693 | Referent  0.4 (0.1-1.3) |  | 1531 | Referent  0.3 (0.0-2.4) |
|  | FGF2R (C>T) |  | CC  CT  TT | 227  251  79 | 185  207  57 | Referent   1. (0.8-1.3)   0.9 (0.6-1.3) |  | 153  170  51 | Referent  1.0 (0.8-1.4)  1.0 (0.6-1.5) |  | 60  69  26 | Referent  1.0 (0.7-1.5)  1.3 (0.8-2.3) |
|  |  | Dominant | CC  T carriers | 227  330 | 185  264 | Referent  1.0 (0.8-1.3) |  | 153  221 | Referent  1.0 (0.8-1.3) |  | 60  95 | Referent  1.1 (0.7-1.6) |
|  |  | Recessive | C carriers  TT | 478  79 | 392  57 | Referent  0.9 (0.6-1.3) |  | 323  51 | Referent  1.0 (0.7-1.4) |  | 129  26 | Referent  1.3 (0.8-2.1) |
| Tumoral necrosis factor alpha | TNFA-308 G>A |  | GG  GA  AA | 407  143  7 | 326  115  8 | Referent  1.0 (0.7-1.3)  1.3 (0.5-3.8) |  | 277  89  8 | Referent  1.0 (0.7-1.2)  1.6 (0.6-4.4) |  | 110  41  4 | Referent  1.0 (0.6-1.5)  1.9 (0.5-6.6) |
|  |  | Dominant | GG  A carriers | 407  150 | 326  123 | Referent  1.0 (0.8-1.3) |  | 277  97 | Referent  1.0 (0.7-1.3) |  | 110  45 | Referent  1.0 (0.7-1.6) |
|  |  | Recessive | G carriers  AA | 550  7 | 441  8 | Referent  1.3 (0.5-3.7) |  | 366  8 | Referent  1.6 (0.6-4.5) |  | 151  4 | Referent  1.9 (0.5-6.6) |
|  | TNFA-863 C>A |  | CC  CA  AA | 353  171  32 | 289  141  18 | Referent  1.0 (0.8-1.3)  0.7 (0.4-1.2) |  | 238  119  16 | Referent  1.0 (0.8-1.4)  0.7 (0.4-1.4) |  | 93  55  6 | Referent  1.2 (0.8-1.8)  0.7 (0.3-1.7) |
|  |  | Dominant | CC  A carriers | 353  203 | 289  159 | Referent  1.0 (0.7-1.2) |  | 238  135 | Referent  1.0 (0.7-1.3) |  | 93  61 | Referent  1.1 (0.8-1.6) |
|  |  | Recessive | C carriers  AA | 524  32 | 430  18 | Referent  0.7 (0.4-1.2) |  | 357  16 | Referent  0.7 (0.4-1.4) |  | 148  6 | Referent  0.6 (0.3-1.6) |

Supplementary table II. Age-adjusted Odds Ratios and 95%CI of prostate cancer according to adipokine pathways polymorphisms (cont´d)

|  |  |  |  |  | Age-adjusted Odds Ratios | | | | | | | |
| --- | --- | --- | --- | --- | --- | --- | --- | --- | --- | --- | --- | --- |
|  |  |  |  | Non-PCa | All PCa | |  | Restricted to HGPCa ^a^ | |  | Restricted to HRPCaM ^b^ | |
| Pathway | SNP | Model | Genotypes | N | N | OR (95% CI) |  | N | OR (95% CI) |  | N | OR (95% CI) |
| Tumoral necrosis factor alpha | TNFRSF1A-329 G>T |  | GG  GT  TT | 203  275  78 | 155  228  66 | Referent  1.1 (0.8-1.4)  1.1 (0.8-1.7) |  | 130  191  53 | Referent  1.1 (0.8-1.4)  1.1 (0.7-1.7) |  | 58  73  24 | Referent  0.9 (0.6-1.3)  1.1 (0.6-2.0) |
|  |  | Dominant | GG  T carriers | 203  353 | 155  294 | Referent  1.1 (0.8-1.4) |  | 130  244 | Referent  1.1 (0.8-1.4) |  | 58  97 | Referent  0.9 (0.6-1.3) |
|  |  | Recessive | G carriers  TT | 478  78 | 383  66 | Referent  1.1 (0.8-1.6) |  | 321  53 | Referent  1.1 (0.7-1.6) |  | 131  24 | Referent  1.2 (0.7-2.0) |

N, number of evaluable patients; SNP, single nucleotide polymorphism; OR (95%CI), age-adjusted odds-ratio and respective 95% confidence interval

^a^ HGPCa,High-grade Prostate Cancer (Gleason grade ≥7)

^b^ HRPCaM, High-risk Prostate Cancer for metastasis (Gleason grade ≥ 8 and/or PSA ≥ 20 ng.mL^-1^)
