# Supplementary material for: Performance of an Adipokine Pathway-Based Multilocus Genetic Risk Score for Prostate Cancer Risk Prediction
Source: PLoS One. 2012 Jun 29;7(6):e39236. doi: 10.1371/journal.pone.0039236 (PMC3387135; doi:10.1371/journal.pone.0039236)
Supplement: Table S3 — Age-adjusted Odds Ratios and 95%CI for prostate cancer (PCa) associated with selected SNPs, after age stratification. a High-grade Prostate Cancer, Gleason grade ≥7; b High-risk Prostate Cancer for metastasis, Gleason grade ≥8 and/or PSA ≥20 ng/mL; aOR (95%CI), age-adjusted odds ratio and respective 95% Confidence Interval; PCa, Prostate Cancer; Median age at diagnosis = 67.5 years; *Evaluable individuals for analysis. (DOC) [file pone.0039236.s003.doc]

Table S3. Age-adjusted Odds Ratios and 95%CI for prostate cancer (PCa) associated with selected *SNPs,* after age stratification

|  | Age | Non-PCa | All PCa | |  | Restricted to High-grade PCa ^a^ | |  | Restricted to High-risk PCa for metastasis ^b^ | |
| --- | --- | --- | --- | --- | --- | --- | --- | --- | --- | --- |
| SNPs | Group | N * | N | aOR (95%CI) |  | N * | aOR (95%CI) |  | N * | aOR (95%CI) |
| *LEPR* Gln223Arg AA | < Median | 276 | 163 | 1.7(1.1-2.6) |  | 126 | 1.8(1.1-2.8) |  | 42 | 2.3(1.2-4.4) |
|  | ≥ Median | 277 | 284 | 1.5(1.1-2.2) |  | 247 | 1.6(1.1-2.3) |  | 112 | 1.7(1.0-2.7) |
| *IL6R* Asp358Ala C carriers | < Median | 276 | 163 | 1.8(1.2-2.7) |  | 126 | 1.9(1.2-2.9) |  | 42 | 3.4(1.5-7.5) |
|  | ≥ Median | 277 | 284 | 0.9(0.6-1.3) |  | 247 | 1.0(0.7-1.4) |  | 112 | 0.9(0.6-1.5) |
| *IL6*-597 G carriers | < Median | 276 | 163 | 1.2(0.7-2.2) |  | 126 | 1.3(0.7-2.6) |  | 42 | 2.0(0.6-6.6) |
|  | ≥ Median | 277 | 284 | 1.5(0.9-2.5) |  | 247 | 1.6(0.9-2.9) |  | 112 | 1.0(0.5-1.9) |
| *OPN* -66 GG | < Median | 276 | 163 | 2.1(1.0-4.4) |  | 126 | 2.3 (1.1-4.9) |  | 42 | 3.5(1.3-9.1) |
|  | ≥ Median | 277 | 284 | 1.5(0.7-3.2) |  | 247 | 1.5(0.7-3.2) |  | 112 | 1.8(0.7-4.5) |
| *IGFBP3* -202 CC | < Median | 276 | 163 | 1.6(1.0-2.6) |  | 126 | 1.6(1.0-2.6) |  | 42 | 1.1(0.5-2.4) |
|  | ≥ Median | 277 | 284 | 1.1(0.8-1.6) |  | 247 | 1.2(0.8-1.7) |  | 112 | 1.1(0.6-1.8) |
| *IGF1R* +3174 AA | < Median | 276 | 163 | 1.6(1.0-2.6) |  | 126 | 1.7(1.0-2.7) |  | 42 | 1.7(0.8-3.6) |
|  | ≥ Median | 277 | 284 | 1.1(0.7-1.8) |  | 247 | 1.1(0.7-1.9) |  | 112 | 1.0(0.5-2.0) |
| *FGF2* +223 CC | < Median | 276 | 163 | 1.4(0.8-2.3) |  | 126 | 1.3(0.8-2.4) |  | 42 | 1.4(0.5-3.4) |
|  | ≥ Median | 277 | 283 | 1.3(0.8-2.1) |  | 246 | 1.4(0.8-2.4) |  | 111 | 1.6(0.8-3.4) |

^a^ High-grade Prostate Cancer, Gleason grade ≥7; ^b^ High-risk Prostate Cancer for metastasis, Gleason grade ≥ 8 and/or PSA ≥ 20 ng/mL; aOR (95%CI), age-adjusted odds ratio and respective 95% Confidence Interval; PCa, Prostate Cancer; Median age at diagnosis=67.5 years; * Evaluable individuals for analysis
