# Supplementary material for: Performance of an Adipokine Pathway-Based Multilocus Genetic Risk Score for Prostate Cancer Risk Prediction
Source: PLoS One. 2012 Jun 29;7(6):e39236. doi: 10.1371/journal.pone.0039236 (PMC3387135; doi:10.1371/journal.pone.0039236)
Supplement: Table S4 — Sensitivity analysis in SNPs with deviation from Hardy-Weinberg equilibrium. Risk for prostate cancer after restriction on the non-prostate cancer group to just benign prostate hyperplasia and normal or to PSA below 4 ng/mL. *Hardy-Weinberg equilibrium, Pearson chi-square analysis for differences between observed and expected genotype frequencies; **Age-adjusted odds ratios; BPH, Benign Prostate Hyperplasia; PSA, Prostate-specific Antigen; PSA, prostate-specific antigen; SNP, signle nucleotide polymorphism; aOR (95%CI), age-adjusted odds ratio and respective 95% confidence interval. a Biopsy findings: normal, 14.9%; BPH, 5.4%, chronic prostatitis, 74.7%; atrophy, 5%; b Biopsy findings: normal, 73.5%; BPH, 26.5%; c Biopsy findings: normal, 22.2%; BPH, 6.0%, chronic prostatitis, 65.8%; atrophy, 6.0%. (DOC) [file pone.0039236.s004.doc]

Table S4. Sensitivity analysis in SNPs with deviation from Hardy-Weinberg equilibrium. Risk for prostate cancer after restriction on the non-prostate cancer group to just benign prostate hyperplasia and normal or to PSA below 4 ng/mL

|  | Non-Prostate Cancer Groups | | | | | | | | | | |
| --- | --- | --- | --- | --- | --- | --- | --- | --- | --- | --- | --- |
|  | All Non-Prostate Cancer ^a^ | | |  | Restricted to BPH and Normal ^b^ | | |  | Restricted to PSA < 4 ng.mL^-1 c^ | | |
| Risk genotypes | N | aOR (95%CI) ** | P * |  | N | aOR (95%CI) ** | P * |  | N | aOR (95%CI) ** | P * |
| *LEPR* Gln223Arg AA | 557 | 1.6 (1.2-2.1) | 0.008 |  | 113 | 1.6 (1.0-2.5) | 0.664 |  | 117 | 1.5 (0.9-2.3) | 0.755 |
| *IGF1R* +3174 AA | 557 | 1.3 (1.0-1.9) | 0.006 |  | 113 | 1.4 (0.8-2.6) | 0.770 |  | 117 | 1.4 (0.8-2.5) | 0.306 |
| *FGF2* +223 CC | 556 | 1.4 (1.0-2.0) | 0.0005 |  | 113 | 1.2 (0.7-2.2) | 0.936 |  | 117 | 1.6 (0.9-2.8) | 0.036 |

* Hardy-Weinberg equilibrium, Pearson chi-square analysis for differences between observed and expected genotype frequencies; ** Age-adjusted odds ratios; BPH, Benign Prostate Hyperplasia; PSA, Prostate-specific Antigen; PSA, prostate-specific antigen; SNP, signle nucleotide polymorphism; aOR (95%CI), age-adjusted odds ratio and respective 95% confidence interval.

^a^ Biopsy findings: normal, 14.9%; BPH, 5.4%, chronic prostatitis, 74.7%; atrophy, 5%; ^b^ Biopsy findings: normal, 73.5%; BPH, 26.5%; ^c^ Biopsy findings: normal, 22.2%; BPH, 6.0%, chronic prostatitis, 65.8%; atrophy, 6.0%
